# Pivot Shift Grade and Patient-Reported Outcomes After Repair of Lateral Meniscus Posterior Root Tears Combined With Anterior Cruciate Ligament (ACL) Reconstruction Versus Those After Isolated ACL Reconstruction at Two-Year Follow-Up

**DOI:** 10.7759/cureus.95976

**Published:** 2025-11-03

**Authors:** Yusuke Nakagawa, Aritoshi Yoshihara, Yusuke Matsuyama, Tomomasa Nakamura, Hiroki Katagiri, Nobutake Ozeki, Masaki Amemiya, Ichiro Sekiya, Takeo Fujiwara, Hideyuki Koga

**Affiliations:** 1 Department of Cartilage Regeneration, Institute of Science Tokyo, Tokyo, JPN; 2 Department of Joint Surgery and Sports Medicine, Institute of Science Tokyo, Tokyo, JPN; 3 Department of Global Health, Institute of Science Tokyo, Tokyo, JPN; 4 Department of Joint Surgery and Sports Medicine, Institute of Science Tokyo Hospital, Tokyo, JPN; 5 Department of Orthopaedic Surgery, Institute of Science Tokyo Hospital, Tokyo, JPN; 6 Center for Stem Cell and Regenerative Medicine, Institute of Science Tokyo Hospital, Tokyo, JPN; 7 Center for Stem Cell and Regenerative Medicine, Institute of Science Tokyo, Tokyo, JPN; 8 Department of Joint Surgery and Sports Medicine, Graduate School of Medical and Dental Sciences, Institute of Science Tokyo, Tokyo, JPN

**Keywords:** anterior cruciate ligament, clinical outcomes, lateral meniscus, meniscus repair, posterior root tear

## Abstract

Objectives: Lateral meniscus posterior root tear (LMPRT) is often accompanied by anterior cruciate ligament (ACL) injury and increases both anterolateral rotatory instability and load on the articular cartilage. However, studies on the outcomes of LMPRT repair are scarce. Thus, we aimed to evaluate the clinical outcomes of LMPRT with ACL reconstruction (ACLR).

Methods: This study included 107 patients who underwent primary ACLR using autologous hamstring tendons and were followed up for two years. Twenty-five patients who underwent simultaneous LMPRT repair were classified into the LMPRT group, and 82 patients who underwent isolated ACLR without any meniscal procedures were classified into the isolated ACLR group. Both subjective and objective outcomes were measured before and two years after surgery, and subjective outcomes were also collected at three months and one year post-surgery. The clinical outcomes of the two groups were compared after adjusting for age, sex, body mass index (BMI), pre-injury Tegner scale scores, and time from injury to surgery. Improvement in clinical outcomes was evaluated using repeated analysis of variance (ANOVA).

Results: After adjusting for all covariates, the preoperative pivot shift grade was higher in the LMPRT group than in the ACLR group. Preoperative subjective outcomes were significantly poorer in the LMPRT group than those in the isolated ACLR group, except for the International Knee Documentation Committee score. At the two-year follow-up, the Lysholm score in the LMPRT group was higher than that in the ACLR group. The LMPRT group exhibited a significantly greater improvement in subjective outcomes than that in the isolated ACLR group. Improvements in the objective measurements of the two groups were not significantly different. Although sagittal extrusion increased, the lateral meniscus extrusion (LME) width significantly decreased by an average of 0.4 mm in the LMPRT group before and one year after surgery.

Conclusion: Patients with LMPRT and ACL injuries simultaneously had higher pivot-shift grades and reported worse subjective outcomes prior to surgery compared to those with isolated ACL injuries; however, the clinical outcome of ACLR with LMPRT was not inferior to that of isolated ACLR at two years postoperatively. Following LMPRT repair, the patients were able to restore knee joint stability and showed similar recovery levels as patients with isolated ACLR for at least a short period.

## Introduction

Anterior cruciate ligament (ACL) injuries are often accompanied by meniscus injury. It has been reported that meniscus lesions are found in 16%-82% of primary ACL reconstructions (ACLRs) [1]. The menisci serve a crucial function by demonstrating shock-absorbing and load-spreading capabilities as well as supporting the stability of the knee joint. In ACL-deficient knees, the medial meniscus may function as a secondary stabilizer of anterior tibial translation, while the lateral meniscus may help alleviate anterolateral rotational laxity [2].

A meniscal root tear is an avulsion injury of the meniscotibial ligament or radial tear of the meniscus within 1 cm of the attachment site. In approximately 10%-15% of cases involving ACL injuries, a lateral meniscus posterior root tear (LMPRT) is observed; however, similar root tears on the medial meniscus are far less common [3]. LMPRT influences both knee laxity and tibiofemoral contact pressure in ACL-injured knees, as observed in clinical studies [3] and biomechanical experiments [4,5]. Studies examining the biomechanical effects of LMPRT repair have found improvements in knee stability, decreases in ACL graft force, and restoration of load distribution function [6,7]. However, thus far, there have been a limited number of studies on clinical outcomes post-ACLR with LMPRT repair [8-10]; in particular, no report has evaluated anterolateral rotatory stability following LMPRT repair. Randomized controlled trials (RCTs) are the gold standard for such clinical evaluations; however, it could be ethically difficult not to provide treatment to patients with LMPRT in the control group. Thus, the primary objective of the present study was to compare the anterolateral rotatory stability of patients with combined ACL injury and LMPRT with those with isolated ACL injury before and after surgery for two years. Pivot-shift test grade was selected as the primary outcome. In addition, patient-reported outcomes were designated as secondary outcomes. Other clinical measurements were also compared. We hypothesized that the pivot-shift test grade in patients with ACL injury with LMPRT would be greater than that in patients with isolated ACL injury preoperatively, whereas the differences would become nonsignificant after ACLR concomitant with LMPRT repair.

## Materials and methods

Patients

**Figure 1:**
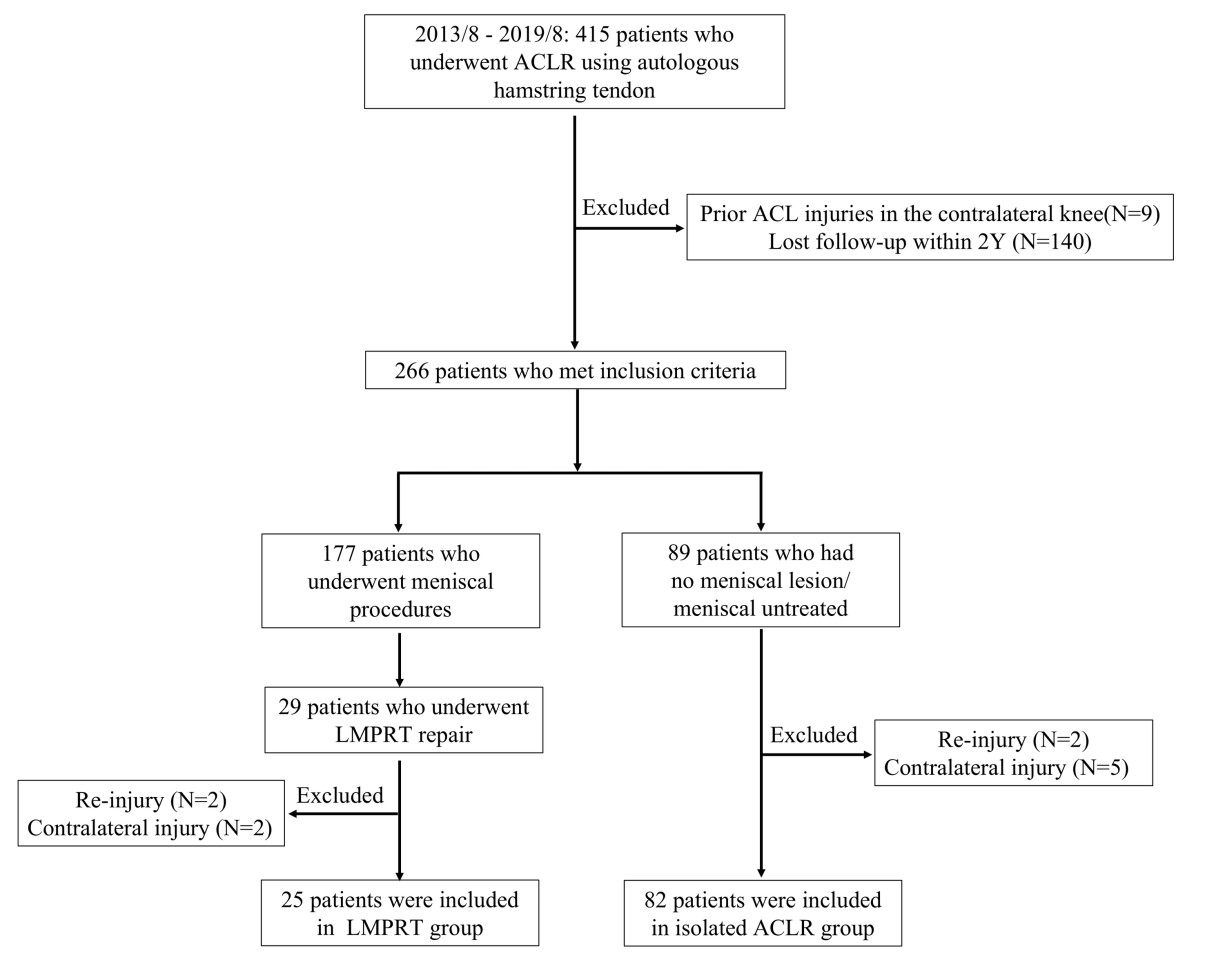
Flowchart showing the selection and grouping of patients for analyses ACLR: Anterior cruciate ligament reconstruction; LMPRT: Lateral meniscus posterior root tear.

This retrospective comparative study was approved by the Institutional Review Board of the Tokyo Medical and Dental University Hospital (research protocol identification number: 2000-1146). Patients who underwent primary ACLR using autologous hamstring tendon(s) between August 2013 and August 2019 were included. Based on the inclusion criteria, 415 patients were identified in a review of our prospectively collected database. Patients with prior injuries or surgeries in the contralateral knee (N = 9) and those who were lost to follow-up within two years (N = 140) were excluded, leaving 266 eligible patients. Among them, 29 patients underwent LMPRT, which required repair. Two patients had ACL re-injury, and two patients had contralateral ACL injury within two years, and they were excluded from further analyses; hence, 25 patients were included in the LMPRT group. Eighty-nine patients had no meniscal lesions or had untreated meniscal lesions. During the two-year follow-up, two patients had ACL re-injury, and five patients had contralateral ACL injury; hence, 82 patients were included in the isolated ACLR group (Figure 1).

Based on the Laprade classification of root tears [11], nine, seven, five, and four patients in the LMPRT group had type 2a, 2b, 2c, and 4 tears, respectively. Two main surgical techniques are applied in LMPRT repair. Radial tears with sufficient remaining tissue of a root remnant were treated using a side-to-side suture repair method. The root avulsions and radial tears of an inadequate meniscal remnant were treated using a pull-out repair technique. Eleven patients in the LMPRT group had medial meniscal injuries. Seven patients had longitudinal tears, one had a horizontal tear, and one had a radial tear, all of which were repaired with an all-inside device. Two patients had bucket handle tears, which were treated with inside-out and all-inside repair. Six patients had other LM injuries that required specific procedures. Four patients had longitudinal tears and underwent inside-out repair. Two patients had flap tears: one was excised, and the other was treated with inside-out repair.

Operative procedures

**Figure 2:**
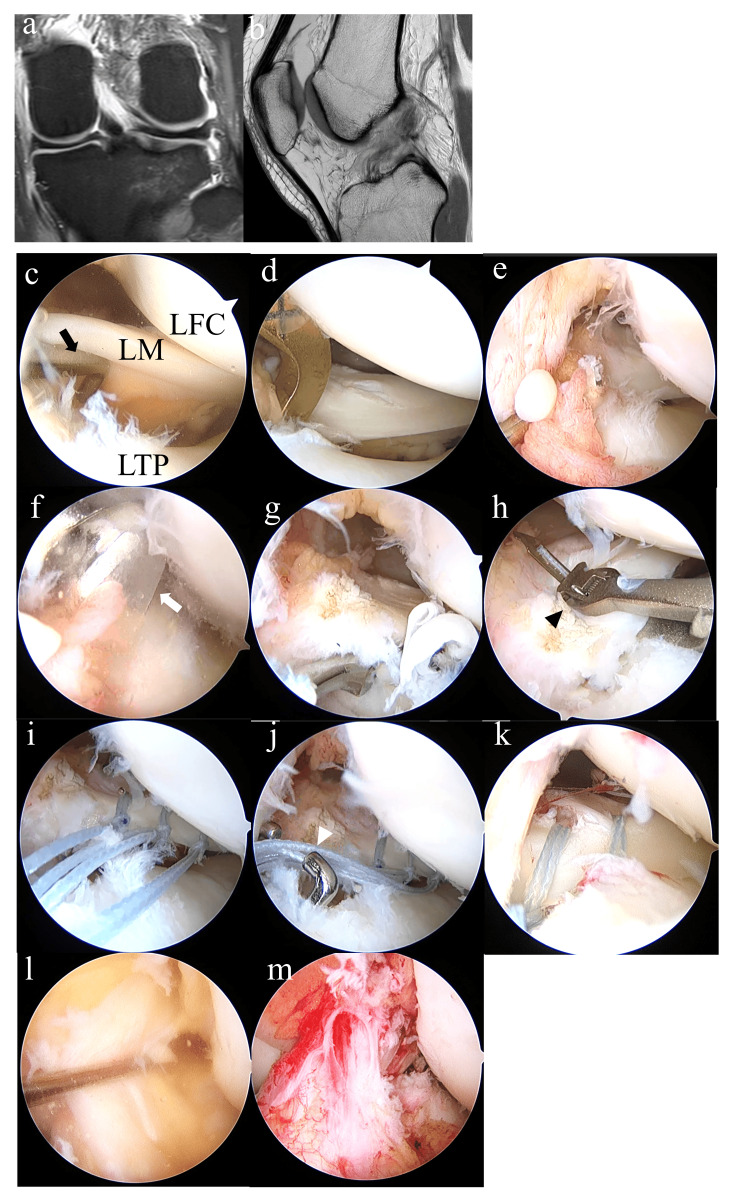
Representative case 1 (a, b) Preoperative MR images. (a) No apparent meniscus injury. (b) Torn ACL. (c-m) Arthroscopic findings. (c) Type 2a LMPRT with positive lift-off sign is observed by probing (arrow). (d) The tibial ACL guide is set at the anatomical attachment site of the LM posterior root. (e) A 2.4-mm guide wire is inserted. (f) The bone tunnel is created with a 6.0-mm cannulated reamer (white arrow). (g) Articular cartilage around the bone tunnel is removed with a curette until subchondral bone is exposed to promote adhesion of the meniscus. (h) A 2-0 strong suture for racking hitch knot is placed in the meniscus using Knee Scorpion Suture Passer (arrowhead). (i) Three racking hitch knot sutures securely hold the torn edge of the meniscus. (j) The sutures are introduced into the tunnel using a suture retriever (arrowhead). (k) After fixation, reattachment of the LM posterior root to the insertion site is confirmed. (l) Torn ACL. (m) Reconstructed ACL with preserved remnant.

**Figure 3:**
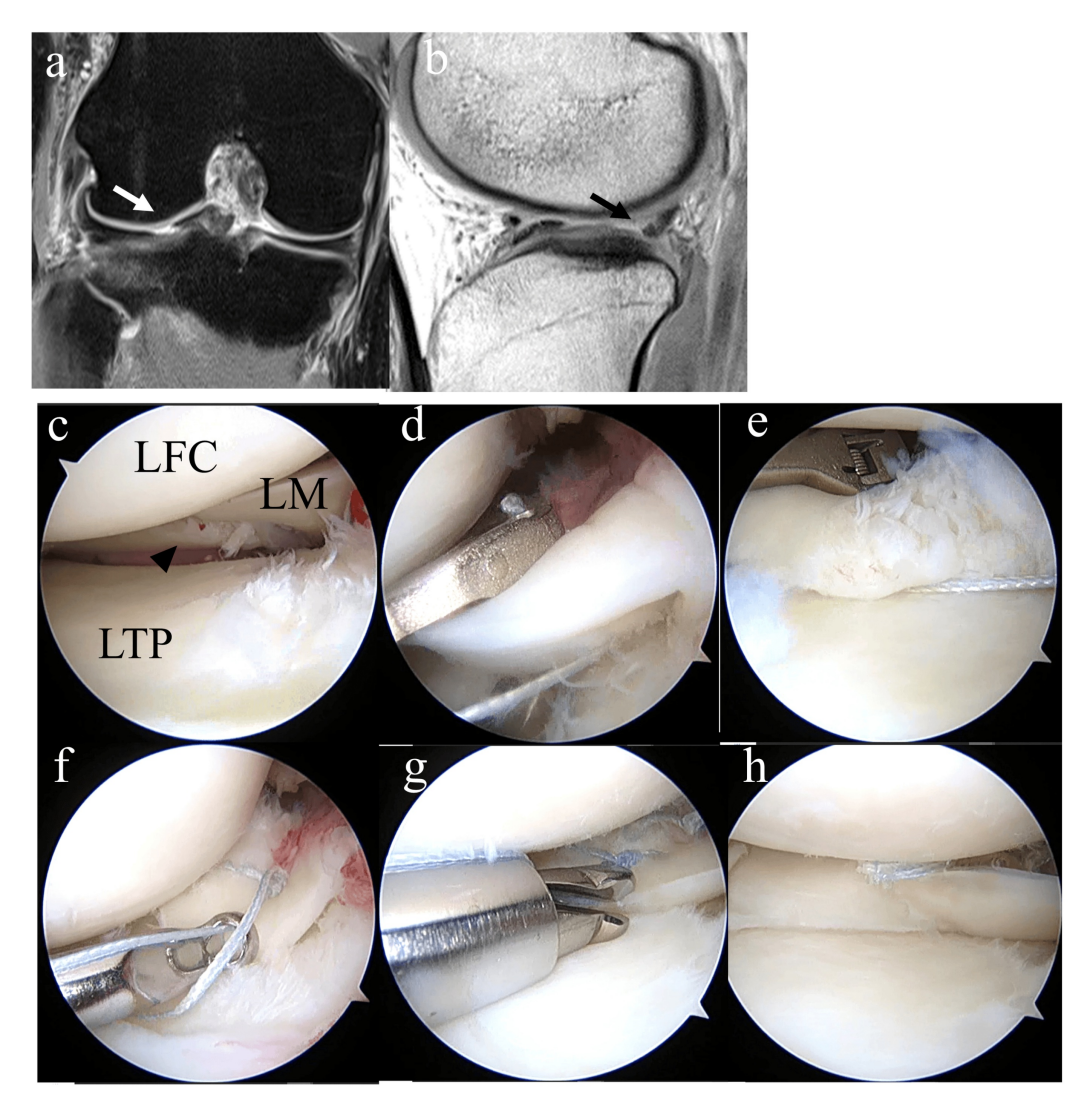
Representative case 2 (a) Preoperative MR image showing a vertical linear defect in the coronal plane to indicate LMPRT (white arrow). (b) Truncated triangle sign (arrow). (c-k) Arthroscopic findings. (c) Type2c LMPRT (arrowhead). (d) One end of a 2-0 FiberWire is placed at one side of the tear using the Knee Scorpion Suture Passer. (e) Another end of the 2-0 FiberWire is placed at another side of the tear to form a horizontal mattress suture. (f) The suture is tied with a sliding knot. (g) The knot is cut with a suture cutter. (h) After two horizontal mattress sutures. MR: Magnetic resonance; ACL: Anterior cruciate ligament; LMPRT: Lateral meniscus posterior root tear; LM: Lateral meniscus; LFC: Lateral femoral condyle; LTP: Lateral tibial plateau.

The operations were either carried out by two attending surgeons or conducted under their direct supervision. A standard arthroscopic procedure was conducted using anterolateral and anteromedial portals to verify the ACL tear and assess the condition of the menisci. If an LMPRT was identified, it was appropriately repaired. The repair techniques and number of sutures needed were decided by each surgeon based on the tear's location, size, and classification. The majority of type 2a and 2b tears were treated with a pull-out procedure (Figure 2) [5,12]; the torn edge of the lateral meniscus posterior root was securely held with two or more 2-0 FiberWires (Arthrex, Naples, FL) by a racking hitch knot. These sutures were passed through an independently created 6-mm-diameter tunnel to the anteromedial aspect of the proximal tibia and then fixed to an anchor staple. For type 2c and type 4 tears, all-inside repair using a Knee Scorpion Suture Passer (Arthrex) or Fast-fix (Smith and Nephew, London, England) was performed (Figure 3).

An oblique 4-cm incision was made on the anteromedial aspect of the tibia at the level of the pes anserinus. The semitendinosus tendon was harvested using an open-loop tendon stripper, and, when necessary, the gracilis tendon was also harvested in the same manner. ACLR was performed using a remnant preserved double-bundle technique with autologous semitendinosus tendon(s) [13]. Femoral and tibial tunnels were drilled at the anatomical locations corresponding to the anteromedial (AM) and posterolateral (PL) bundles' insertion points. The femoral tunnel was established via a technique employing an outside-in or transportal method. A tunnel was drilled from the anteromedial surface of the tibia. Both grafts' femoral sides were secured with the EndoButton CL from Smith and Nephew. Both AM and posteromedial (PM) bundle grafts were secured to an anchor staple with sutures at the tibial site when the knee was flexed at 20 degrees, with the applied initial tension adjusted equal to the per cross-sectional area on the basis of 25 N per 6 mm in diameter.

Postoperative management

The postoperative rehabilitation protocol was the same for all patients, except for the prohibition of weight bearing over 90° of flexion in patients with LMPRT until three months postoperatively. Range-of-motion exercises were initiated three days after surgery, involving full extension to 120 degrees of flexion. Twenty kilograms of partial weight-bearing exercise were permitted after three days, with the weight then gradually increased. The use of crutches was stopped after four weeks. Exercise of the knee muscles was recommended six weeks following surgery, with a focus on closed kinetic movement. Running began at three months, initially as jogging, and subsequently, the pace gradually accelerated. Once 80% of full-speed running was reached, athletic exercises linked to prior sports or desired sporting pursuits were introduced with detailed guidance. Athletic activities were permitted for patients at least six months postoperatively, provided no joint-related adverse symptoms were present, and the patient's muscle recovery had reached or exceeded 90% of the uninjured knee's strength in extension and flexion, as determined by a Cybex machine (Lumex, Ronkonkoma, NY) with a 60/s rate after completion of a specified athletic training regimen.

Clinical evaluations

Both patient-based subjective and objective outcomes were measured before and two years after surgery. Only patient-based subjective outcomes were collected at three months and one year post-surgery. The Lysholm score (it has been open access) [14], International Knee Documentation Committee (IKDC) subjective form (it has been open access) [15], and Knee Injury and Osteoarthritis Outcome Score (KOOS; it has been open access) [16], which consists of five subscales (pain, symptoms, ADL (activity of daily living), sports/recreation, and QOL (quality of life)), were used as patient-based evaluations. Objective evaluations included anterior knee laxity and the pivot-shift test. Anterior knee laxity, measured with the KT-1000 arthrometer (MED metric, San Diego, CA) at manual maximum pull, was expressed as the difference between the injured and uninjured knees in 0.5-mm increments [17]. In the pivot-shift test, the modified IKDC criteria (Grade 0 = negative; Grade 1 = subtle glide, but not negative; Grade 2 = glide, Grade 3 = between grades 2 and 4; Grade 4 = clunk; Grade 5 = between grades 4 and 6; Grade 6 = gross) were used as the grading system to evaluate the pivot-shift phenomenon as the primary outcome in this study. These modified criteria have high inter-observer reliability with an intra-class correlation coefficient of 0.97 (95% confidence interval (CI): 0.94-0.98) [17]. The maximum extension strength (kg) of both knees was measured using a Cybex machine (Lumex, Ronkonkoma, NY) at 60 deg/s. The extensor muscle strength index expressed as a percentage of the strength of the non-operated knee was reported before surgery, and at three months and one year after surgery.

Measurement of lateral meniscus extrusion width

Either 1.5 T or 3.0 T magnetic resonance imaging (MRI) was used. The lateral meniscus extrusion (LME) width was measured on the coronal slice of the proton-weighted image showing maximum extrusion of the LM preoperatively and at one-year follow-up in the LMPRT group. The LME width, defined as the distance from the most peripheral aspect of the meniscus to the tibia’s border, excluding any osteophytes, was measured in 0.1 mm increments. Sagittal extrusion was measured on the sagittal slice of the proton-weighted image as the distance from the inner margin of the anterior horn of the lateral meniscus to the meniscocapsular junction of the posterior horn in the midsagittal plane. Differences between the preoperative and postoperative measurements were calculated.

Statistical analysis

Clinical outcomes preoperatively and at the two-year follow-up were compared between the LMPRT and isolated ACLR groups. Multiple linear regression analyses were performed after adjusting for age, sex, body mass index (BMI), pre-injury Tegner activity scale scores (it has been open access) [14], and time from injury to surgery (classified into two groups: within three months and more than three months). Repeated-measures analysis of variance (ANOVA) was applied to evaluate the difference in postoperative improvement between the two groups. Furthermore, a paired t-test was used to compare LME width both before and one year after the surgery. For all analyses, P-values < 0.05 were considered statistically signiﬁcant. A post-hoc power analysis revealed that with an alpha value of 0.05, the current study achieved a power of 57.9% and 18.9% for the difference in pivot-shift grade preoperatively and two years after surgery, respectively, and 90.8% for the Lysholm score two years after surgery between the groups. Statistical analyses were performed using the STATA 15.0 software (StataCorp, College Station, TX).

## Results

Table 1 shows the patients’ demographic characteristics. There were no significant differences in age, sex, BMI, Tegner scores, or time from injury to surgery between the two groups.

**Table 1:** Patients’ demographic data Values are expressed as mean with standard deviation. Brackets are expressed as minimum and maximum values. The P-value was obtained using the t-test for age, BMI, and Tegner score, and the chi-square test was used to analyze sex and time from injury to surgery. LMPRT: Lateral meniscus posterior root tear; ACLR: Anterior cruciate ligament reconstruction; BMI: Body mass index.

Demographic variables	LMPRT (N = 25)	Isolated ACLR (N = 82)	P-value
Age, years	31.2 (12-58)	27.3 (13-57)	0.084
Sex (Male/Female)	7/18	24/58	0.903
BMI	22.3 ± 2.3	22.7 ± 2.6	0.751
Tegner score	6.5 (3-9)	6.8 (3-9)	0.823
Time from injury to surgery (0-3 months/>3 months)	17/8	51/31	0.598

The clinical outcomes before surgery are shown in Table 2. Preoperatively, the pivot-shift grade was greater in the LMPRT group than that in the ACLR group (P = 0.025). All preoperative subjective scores in the LMPRT group were lower than those in the ACLR group (P < 0.05).

**Table 2:** Clinical outcomes before surgery Values are expressed as mean with standard deviation, except for the pivot-shift grade, which is presented as the median (range). P-values were obtained using the t-test, except for the pivot-shift grade, which was analyzed using the Mann–Whitney U test. LMPRT: Lateral meniscus posterior root tear; ACLR: Anterior cruciate ligament reconstruction; KT: KT-1000 arthrometer; IKDC: International Knee Documentation Committee; KOOS: Knee injury and osteoarthritis outcome score; ADL: Activities of daily living; Rec: Recreation; QOL: Quality of life.

Measurements	LMPRT (N = 25)	Isolated ACLR (N = 82)	P-value
KT measurement	5.5 ± 1.8	5.8 ± 2.5	0.599
Pivot-shift grade	4 (3-6)	4 (0-6)	0.025
Lysholm score	74.4 ± 13.6	82.5 ± 11.4	0.004
IKDC subjective score	61.7 ± 13.2	68.6 ± 13.0	0.048
KOOS symptom	79.5 ± 16.0	87.7 ± 10.6	0.008
Pain	80.2 ± 15.8	88.0 ± 9.9	0.009
ADL	88.6 ± 10.8	95.4 ± 6.5	<0.001
Sports/Rec	42.4 ± 25.6	68.7 ± 21.0	<0.001
QOL	46.9 ± 25.6	61.8 ± 23.3	0.015

At three months, IKDC, KOOS-ADL, and KOOS sports/rec in the LMPRT group were significantly lower than those in the isolated ACLR group (Table 3; P = 0.037, 0.034, and 0.028, respectively); however, these differences were not significant at one year (Table 4).

**Table 3:** Subjective outcomes three months after surgery Values are expressed as mean with standard deviation. P-values were obtained by the t-test. LMPRT: Lateral meniscus posterior root tear; ACLR: Anterior cruciate ligament reconstruction; KT: KT-1000 arthrometer; IKDC: International Knee Documentation Committee; KOOS: Knee injury and osteoarthritis outcome score; ADL: Activities of daily living; Rec: Recreation; QOL: Quality of life.

Measurements	LMPRT (N = 25)	Isolated ACLR (N = 82)	P-value
IKDC subjective score	59.2 ± 13.6	66.1 ± 12.5	0.037
KOOS symptom	76.5 ± 16.1	80.2 ± 13.6	0.311
Pain	80.9 ± 9.8	83.6 ± 13.1	0.379
ADL	88.7 ± 8.5	93.1 ± 8.0	0.034
Sports/Rec	47.1 ± 25.9	62.3 ± 27.0	0.028
QOL	54.5 ± 20.6	60.0 ± 23.1	0.364

**Table 4:** Subjective outcomes one year after surgery Values are expressed as mean with standard deviation. P-values were obtained using the t-test. LMPRT: Lateral meniscus posterior root tear; ACLR: Anterior cruciate ligament reconstruction; KT: KT-1000 arthrometer; IKDC: International Knee Documentation Committee; KOOS: Knee injury and osteoarthritis outcome score; ADL: Activities of daily living; Rec: Recreation; QOL: Quality of life.

Measurements	LMPRT (N = 25)	Isolated ACLR (N = 82)	P-value
Lysholm score	96.8 ± 6.4	95.9 ± 5.4	0.468
IKDC subjective score	87.7 ± 9.6	87.8 ± 12.0	0.947
KOOS symptom	89.1 ± 11.0	91.4 ± 11.7	0.404
Pain	94.0 ± 6.5	93.9 ± 8.0	0.950
ADL	98.0 ± 3.0	97.9 ± 4.9	0.978
Sports/Rec	83.5 ± 14.3	89.0 ± 15.1	0.118
QOL	81.5 ± 16.2	83.3 ± 19.9	0.690

The Lysholm score in the LMPRT group was higher than that in the isolated ACLR group at the two-year follow-up (Table 5; P = 0.040).

**Table 5:** Clinical outcomes two years after surgery Values are expressed as mean with standard deviation, except for the pivot-shift grade, which is presented as the median (range). P-values were obtained using the t-test, except for pivot-shift grade, which was obtained by the Mann–Whitney U test. LMPRT: Lateral meniscus posterior root tear; ACLR: Anterior cruciate ligament reconstruction; KT: KT-1000 arthrometer; IKDC: International Knee Documentation Committee; KOOS: Knee injury and osteoarthritis outcome score; ADL: Activities of daily living; Rec: Recreation; QOL: Quality of life.

Measurements	LMPRT (N = 25)	Isolated ACLR (N = 82)	P-value
KT measurement	0.4 ± 1.2	0.4 ± 1.3	0.886
Pivot-shift grade	0 (0-3)	0 (0-4)	0.319
Lysholm score	99.0 ± 2.1	96.6 ± 5.4	0.040
IKDC subjective score	93.1 ± 9.7	90.5 ± 11.2	0.326
KOOS symptom	92.6 ± 8.4	94.3 ± 9.0	0.412
Pain	97.4 ± 2.9	94.6 ± 9.2	0.143
ADL	99.6 ± 1.0	98.3 ± 4.5	0.168
Sports/Rec	94.8 ± 6.3	91.7 ± 12.7	0.255
QOL	88.0 ± 15.2	86.9 ± 16.3	0.769

The differences in the outcomes after covariate adjustment are shown in Table 6. The preoperative pivot-shift grade was 0.6 points greater in the LMPRT group than that in the isolated ACL group (95% CI: 0.1-1.0, P = 0.019). The results of other preoperative measurements also did not change after adjustment. Two years after surgery, Lysholm, IKDC, KOOS pain, and KOOS sports/rec in the LMPRT group were significantly higher than those in the isolated ACL group.

**Table 6:** Multivariate linear regression analysis of pre- and postoperative clinical outcomes on lateral meniscus posterior root tear (LMPRT) P-values were obtained using multiple regression analysis adjusted for age, sex, BMI, Tegner score, and time from injury to surgery. LMPRT: Lateral meniscus posterior root tear; ACLR: Anterior cruciate ligament reconstruction; KT: KT-1000 arthrometer; IKDC: International Knee Documentation Committee; KOOS: Knee injury and osteoarthritis outcome score; ADL: Activities of daily living; Rec: Recreation; QOL: Quality of life; CI: Confidence interval.

Dependent variables	Coefficient of LMPRT	95%CI	P-value
Preoperative measurements			
KT measurement	-0.4	-1.5, 0.8	0.520
Pivot-shift test	0.6	0.1, 1.0	0.019
Lysholm	-7.8	-13.4, -2.2	0.007
IKDC subjective score	-7.3	-13.9, 0.6	0.033
KOOS symptom	-7.6	-13.2, -1.9	0.010
Pain	-6.5	-12.0, -0.9	0.022
ADL	-6.6	-10.3, -2.9	0.001
Sports/Rec	-24.4	-34.9, -13.8	<0.001
QOL	-12.2	-23.2, -1.2	0.031
Postoperative measurements			
KT measurement	-0.2	-0.8, 0.4	0.597
Pivot-shift test	0.1	-0.3, 0.5	0.681
Lysholm	2.4	0.3, 4.5	0.028
IKDC subjective score	4.8	0.2, 9.4	0.040
KOOS symptom	-0.1	-4.1, 3.9	0.944
Pain	4.0	0.3, 7.7	0.034
ADL	1.8	-0.1, 3.5	0.059
Sports/Rec	5.0	0.3, 9.7	0.037
QOL	3.6	-3.5, 10.7	0.317

**Figure 4:**
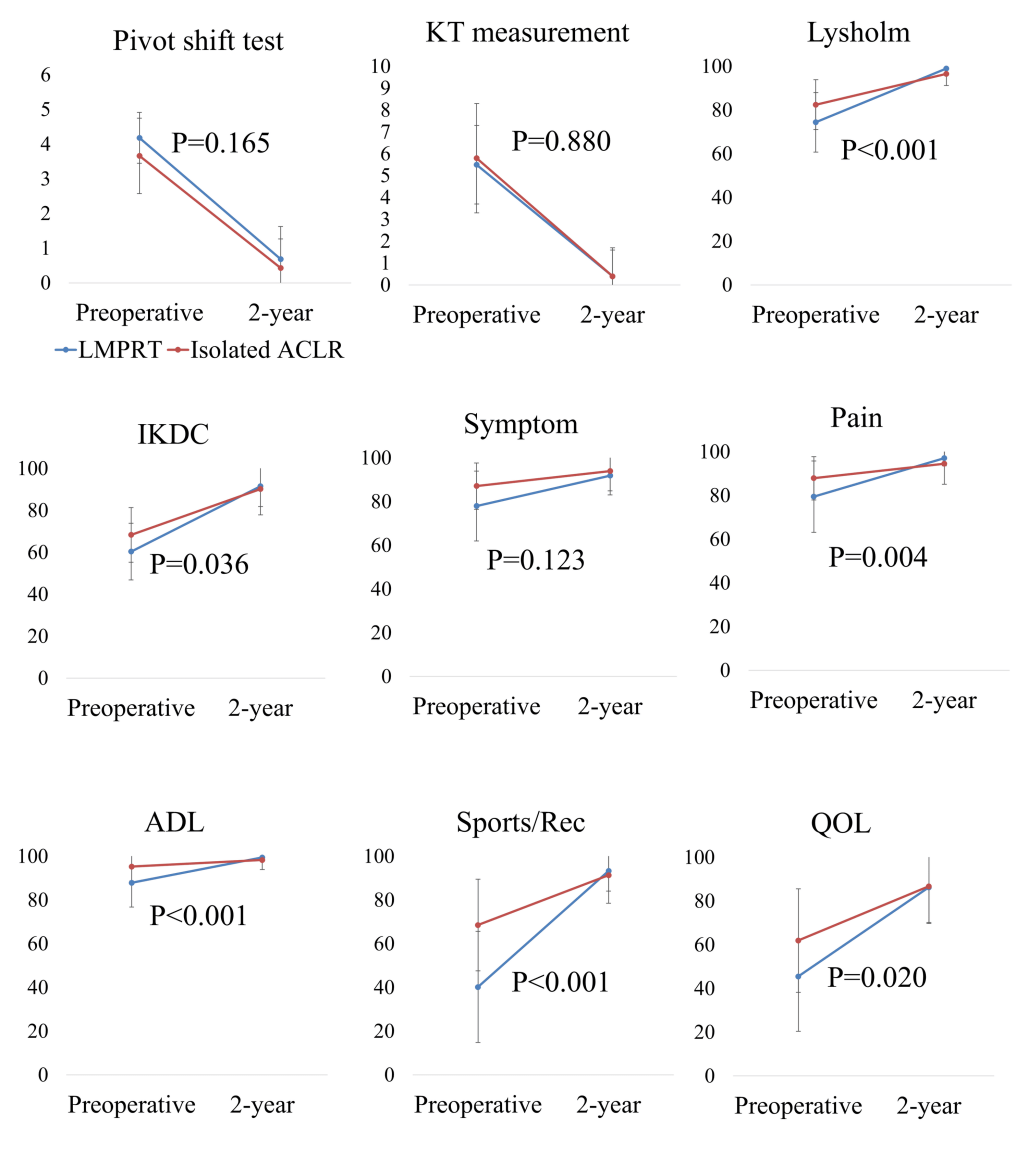
Improvement of subjective outcomes at the two-year follow-up Average preoperative and postoperative clinical outcomes in patients with and without LMPRT. Blue plot and line indicate the LMPRT group. Red plot and line indicate the isolated ACLR group. The P-value, provided using repeated ANOVA, indicates the significance of the interaction term between time and with/without LMPRT. LMPRT: Lateral meniscus posterior root tear; ACLR: Anterior cruciate ligament reconstruction; ANOVA: Analysis of variance.

Repeated ANOVA measurements (Figure 4) showed that improvement in pivot-shift grade was not significantly different between patients with or without LMPRT, while patients with LMPRT exhibited significantly larger improvement in subjective outcomes than those with isolated ACL (P < 0.05), except for the KOOS symptom subscale (P = 0.123).

Extensor muscle strength in the LMPRT group was significantly lower than that in the isolated ACLR group preoperatively and at three months postoperatively, whereas no significant difference was observed between the groups at one year (Table 7).

**Table 7:** Extension muscle strength The extensor muscle strength index was expressed as a percentage of the strength of the non-operated knee. Values are expressed as mean with standard deviation. P-values were obtained using the t-test. LMPRT: Lateral meniscus posterior root tear; ACLR: Anterior cruciate ligament reconstruction.

Time points	LMPRT (N = 25)	Isolated ACLR (N = 82)	P-value
Preoperative	69.6 ± 19.6	86.3 ± 20.5	0.001
3 months postoperative	58.2 ± 20.2	69.3 ± 17.9	0.033
1 year postoperative	84.2 ± 11.8	91.1 ± 16.0	0.060

The LME width significantly decreased by an average of 0.4 mm in the LMPRT group (N = 20; 1.6 ± 0.7 mm vs. 1.2 ± 0.7 mm; P = 0.015), whereas sagittal extrusion increased postoperatively by an average of 1.3 mm.

## Discussion

The most important finding of this study was that preoperative anterolateral rotatory instability, measured with the pivot-shift test, and subjective outcomes were significantly poorer in patients with LMPRT than in those with isolated ACL, while no significant differences were observed other than the Lysholm score two years postoperatively. Patients with LMPRT exhibited significantly larger improvements in subjective outcomes than those with isolated ACL. According to our results, the outcomes of patients with ACL with concomitant LMPRT were comparable to those of patients with isolated ACL after appropriate LMPRT repair during a short-term follow-up; however, this result should be interpreted with caution due to the limited sample size and potential for a type II error.

Advanced age, female sex, higher BMI, lower Tegner scores, and longer time from injury to surgery were associated with poorer preoperative subjective outcomes [18,19]. These factors are also associated with unfavorable clinical outcomes of ACLR during the short-term and long-term follow-up [20]. In the current study, after adjusting for these factors as covariates, LMPRT was found to be associated with poorer subjective outcomes preoperatively. This result suggests that the LMPRT itself can decrease knee function in patients with ACL injuries.

Our multivariable analysis showed that preoperative pivot-shift grade was greater in LMPRT patients than in patients with isolated ACL, whereas anterior-posterior laxity was not significantly different between the two groups. This finding is supported by the fact that the LM plays an important role as a secondary stabilizer to anterolateral rotatory instability in knees with ACL injury [2]. Furthermore, this result corroborated the results of previous biomechanical studies, which reported that anterolateral rotatory instability was executed by LMPRT in ACL-deficient knees [4]. In a clinical study, patients with ACL with LMPRT had a greater pivot-shift grade than patients with isolated ACL without any meniscus lesions preoperatively [3].

The treatment of LMPRT has received more attention among surgeons who perform ACLR because several biomechanical studies have revealed the efficacy of LMPRT repair in decreasing the tibiofemoral contact pressure and restoring the kinematics of the knee joint [5,21]. Tang et al. have recently found that LMPRT repair results in improved anterolateral rotatory stability and decreased ACL graft force in ACL-reconstructed knee settings [7]. A similar finding has been confirmed in several clinical studies. Ahn et al. reported favorable short-term results of all-inside side-to-side LMPRT repair for patients who had undergone ACLR. Significant improvements were observed in the mean subjective IKDC and Lysholm scores, which increased from 67 to 90 and 62 to 93, respectively, with a mean follow-up of 18 months [8]. Anderson et al. documented the clinical outcomes of both all-inside side-to-side repair and transtibial pull-out repair with a mean follow-up period of 58 months in patients who underwent ACLR. For patients who had undergone side-to-side repair, their average subjective IKDC and Lysholm scores were 82 and 87, respectively. In contrast, patients who underwent transtibial pull-out repair had scores of 84 and 86 [10]. Recently, several articles have reported on the clinical consequences of LMPRT, including the subjective outcomes [22,23]. Compared with previous studies, the clinical outcomes in the LMPRT group seem to be favorable regarding patients’ subjective evaluations (Table 8).

**Table 8:** Published patient subjective outcomes of LMPRT repair LMPRT: Lateral meniscus posterior root tear; IKDC: International Knee Documentation Committee; KOOS: Knee injury and osteoarthritis outcome score; ADL: Activities of daily living; Rec: Recreation; QOL: Quality of life.

Measurements	This study (N = 25)	Aga et al. (N = 18) [22]	Shekhar et al. (N = 25) [23]	Ahn et al. (N = 25) [8]	Andersen et al. (N = 16) [10]
Follow-up	24 months	25 months	37 months	41 months	54 months
IKDC subjective score	93.1 ± 2.1	Not available	81.8 ± 11.5	90	84.3 ± 17.0
KOOS symptom	92.6 ± 8.4	79.2 ± 15.1	Not analyzed	Not analyzed	Not analyzed
Pain	97.4 ± 2.9	83.3 ± 15.8	Not analyzed	Not analyzed	Not analyzed
ADL	99.6 ± 1.0	91.9 ± 11.1	Not analyzed	Not analyzed	Not analyzed
Sports/Rec	94.8 ± 6.3	59.7 ± 17.1	Not analyzed	Not analyzed	Not analyzed
QOL	88.0 ± 15.2	60.7 ± 23.7	Not analyzed	Not analyzed	Not analyzed

However, these studies were case series and were not able to show the effects of LMPRT repair itself. In contrast, Pan et al. found that ACLR patients who received LMPRT repair experienced greater functional improvement, although this improvement was not statistically significant and also showed lower rates of radiological osteoarthritis progression in comparison to those who did not have the LMPRT repair [9]. In the present study, we were not able to evaluate the effects of LMPRT repair when compared with patients whose LMPRT was resected or left untreated. Instead, patients who did not undergo meniscal procedures were used as the “control group.” We observed that the anterolateral rotatory stability and subjective outcomes of the patients who received LMPRT repair improved to the same level as those of the control group. This suggests that LMPRT repair could restore meniscus function to a normal level for at least two years. In a recent study, LM repair contributed to anterolateral rotatory stability, as indicated by reduced tibial acceleration during the pivot-shift test measured with a triaxial accelerometer during ACLR surgery [24]. Although meniscus procedures may not influence patient subjective outcomes at short-term follow-up [25], the benefit of meniscus repair has been shown by several long-term follow-up studies [26]. To determine the effectiveness of LMPRT repair, further research involving long-term follow-up and image analysis to assess osteoarthritis change would be necessary.

In the present study, extensor muscle strength in the LMPRT group was significantly lower than that in the isolated ACLR group preoperatively and at three months postoperatively, but no significant difference was observed between the groups at one year. The preoperative weakness in the LMPRT group may reflect a greater degree of initial knee injury and pain associated with the meniscal root tear and other concomitant meniscal injuries, which could have limited voluntary quadriceps activation before surgery. The delayed recovery of extensor strength at three months may also be related to the greater surgical invasiveness of meniscal repair, even though the rehabilitation protocols were almost identical between the two groups. Nevertheless, the comparable muscle strength at one year suggests that LMPRT repair does not impair long-term quadriceps recovery after ACL reconstruction. These findings indicate that temporary weakness during the early postoperative phase should be anticipated, but full recovery can be expected with appropriate rehabilitation.

None of the patients required subsequent procedures for re-tear of the LMPRT. The blood supply to the meniscus root is more abundant than that to the meniscus body, which enhances meniscal healing after repair [27]. Moreover, meniscus repair combined with ACLR leads to better healing than isolated meniscus repair. Several studies have reported that the healing rate of LMPRT after repair and the complete/partial healing rate determined using second-look arthroscopy or MRI ranged from 86.7% to 100% [8,10,28,29], which seemed to be better than the 61% healing rate of radial tears in the midbody of LM [30]. In this study, LME width was significantly reduced by an average of 0.4 mm after LMPRT repair, which is in line with previous studies. Tsuji et al. reported an average 0.16 mm decrease in LME with LMPRT repair [29]. Ahn et al. also showed that LME was reduced by an average of 0.39 mm after side-by-side repair of the LMPRT, despite the difference not being statistically significant [8]. This reduction in LME width may reflect the restoration of the hoop tension and meniscal integrity achieved by the root repair, which helps normalize the lateral compartment contact mechanics. Regarding sagittal extrusion, the present study showed an average increase of 1.3 mm, which is comparable to the 1.2 mm increase reported by Tsujii et al. [29]. The postoperative increase in sagittal extrusion observed in both studies may be attributed to partial healing or incomplete restoration of meniscocapsular attachment in some cases. Although such sagittal extrusion is often observed even after anatomically successful LMPRT repair, its clinical relevance remains uncertain. Longer follow-up studies are warranted to determine whether these morphologic changes on MRI are associated with long-term alterations in articular cartilage status or functional outcomes.

Limitations

The present study has several limitations. First, the patients were from a single institution, and the sample size was relatively small. The post-hoc power analysis indicated an 18.9% postoperative difference in the pivot-shift test. Thus, it should be noted that the nonsignificant difference between the postoperative pivot-shift grades of the two groups might be a false negative due to the limited sample size. In addition, as this was a single-center study, the results should be carefully interpreted in other settings. Second, the follow-up period was short, and the repaired meniscus was not evaluated using MRI or second-look arthroscopy. Third, clinical evaluations were conducted without blinding of the assessors. Lastly, 11 of the 25 patients in the LMPRT group underwent medial meniscus procedures, and six patients had other injuries in the LM that required other procedures. These meniscal procedures, other than LMPRT repair, may have affected the clinical outcomes. Despite these limitations, our results provide some information on the appropriate management of LMPRT during ACLR.

## Conclusions

Patients with concomitant LMPRT and ACL injury showed a greater pivot-shift grade and poorer subjective outcomes than patients with isolated ACL preoperatively. Although no significant difference in the pivot-shift grade or patient-reported outcomes was detected between the LMPRT repair group and the isolated ACL reconstruction group at two years, the interpretation of this result should be made cautiously given the limited sample size. After LMPRT repair, the patients were able to restore knee joint stability and showed similar recovery levels as isolated ACLR patients for at least a short period.
